# Effect of Powder Bed Fusion Laser Sintering on Dimensional Accuracy and Tensile Properties of Reused Polyamide 11

**DOI:** 10.3390/polym15234602

**Published:** 2023-12-02

**Authors:** Urvashi F. Gunputh, Gavin Williams, Marzena Pawlik, Yiling Lu, Paul Wood

**Affiliations:** College of Science and Engineering, University of Derby, Quaker Way, Derby DE22 1GB, UK; g.williams1@derby.ac.uk (G.W.); m.pawlik@derby.ac.uk (M.P.); y.lu@derby.ac.uk (Y.L.); p.wood7@derby.ac.uk (P.W.)

**Keywords:** powder bed fusion laser sintering, polyamide 11, reuse, dimensional accuracy, tensile properties, crystallinity

## Abstract

Polyamide 11 (PA11) is a plant-based nylon made from castor beans. Powder bed fusion laser sintering (PBF-LS) is an additive manufacturing process used for PA11 which allows for the reuse of the unsintered powder. The unsintered powder is mixed with virgin powders at different refresh rates, a process which has been studied extensively for most semi-crystalline polyamides. However, there is lack of information on the effect of using 100% reused PA11 powder and the effect of the number of times it is reused on its own, during powder bed fusion laser sintering. This paper investigates the effect of reusing PA11 powder in PBF-LS and the effect of the number of times it is reused on the dimensional accuracy, density and thermal and tensile properties. From the 100% virgin powder to the third reuse of the powder, there is a decrease in powder wastage, crystallinity and tensile strength. These are associated with the polymerisation and cross-linking process of polymer chains, upon exposure to high temperatures. This results in a higher molecular weight and, hence, a higher density. From the fourth reuse to the tenth reuse, the opposite is observed, which is associated with an increase in high-viscosity unmolten particles, resulting in defects in the PBF-LS parts.

## 1. Introduction

Powder bed fusion laser sintering, PBF-LS, is a powder-based additive manufacturing process that uses a laser beam to sinter the powder particles layer by layer. The surrounding unmolten powder supports the 3D-printed part, and, hence, there is no need for support structures. However, if the unmolten powder feedstock is not used, it must be disposed of, which is wasteful, time-consuming, and expensive [1,2]. Reusing the powder from the PBF-LS process is the solution, although material properties have been shown to deteriorate with this practice [3,4,5,6]. Polymers are the most commonly used material for PBF-LS [7], with polyamides being the leading thermoplastics in use [8]. Polyamide 12 (PA12) is the most widely employed polyamide, due to its low cost, ease of processability, high mechanical strength [8] and high melt temperature compared to its crystallisation temperature [9]. Nonetheless, it is petroleum-based, and the reuse of this powder is still associated with a certain amount of carbon dioxide emissions [6]. Polyamide 11 is a plant-based nylon made from castor beans, which has high strength, durability and resistance to chemicals and heat [10]. When compared to PA12, it has been shown to have a higher toughness and ductility, at the expense of a slightly lower modulus and a higher hydrothermal ageing resistance [11]. With the aim of sustainable manufacturing, there is an increasing interest in the PBF-LS of PA11. The use of this powder for various applications, including medical device manufacturing, has been investigated [12,13] and understanding its reuse will add to the standards associated with medical device manufacturing, using powder-based AM processes such as ASTM F3456-22 [14]. 

Polyamides are semi-crystalline; that is, they have both amorphous (irregularly arranged polymer chains) and crystalline (regularly arranged polymer chains) phases. The effect of reusing polyamide powder during PBF-LS process will be dependent on how the different phases react to repeated exposure to high temperatures. Heat flow maps, created using a differential scanning calorimetry (DSC) method, have been used extensively to characterise the melting/crystallisation of PBF-LS sintered parts, which reflect on the polymer chains’ reactions to heating and cooling down [4,5,15]. Using such heat flow maps, virgin PA12 and polyamide 6 (PA6) sintered parts were found to contain 47% crystalline phase compared to amorphous [16]. Reusing such polyamide powders (without mixing them with fresh powder) reduced the crystallinity of the sintered parts, which significantly reduced their ultimate tensile strength (UTS), elongation at break and maximum shear stress [5]. This was associated with an increase in molecular weight with reuse, caused by the post-condensation reaction during the polymerisation of the polyamide [15]. This increase in weight reduces the mobility of the polymer chains and affects the sintering process adversely. Conversely, Yao et al. [17] associated a lower density with the reuse of PA2200 powder during an PBF-LS process. This reduction in density was related to the presence of voids in the sintered part, which in turn caused the deterioration of the mechanical properties of the latter [17]. Composites of virgin PA11 (fresh and not mixed with reused powder), targeted for the PBF-LS process, exhibited 38% crystallinity [18], while virgin PA11 powder, on its own, exhibited approximately 30% crystallinity [19]. Virgin PA11 powder, targeted for a multi jet fusion (MJF) process, was shown to have a crystallinity of 39%, while its 3D-printed part had a crystallinity of 26% [20]. The resulting MJF PA11 part had an UTS of 46 MPa and a modulus of 932 MPa. However, this process is different from PBF-LS, whereby there is the use of an ink agent and an infra-red lamp for the heating and fusing of powders [20]. Compression-moulded sheets of PA11 have a crystal fraction of 16% [21]. MJF PA11 has a higher crystallinity than compression-moulded PA11. There is a lack of information on the effect of reusing PA11 in a PBF-LS process on its mechanical properties, which creates the need to investigate reused PBF-LS PA11 parts.

Furthermore, dimensional accuracy is a major issue with powder bed fusion laser sintering [22]. It has been associated with post-process cooling, resulting in shrinkage/warpage [23,24] and high surface roughness, also known as the orange peel effect, owing to unmolten powder at the edges [4,25]. This has been observed with reused PA12 [4,26] whereby the ageing of the latter powders has been associated with the change in powder shape and morphology, which causes powder aggregation, expansion and shrinkage. Yusoff et al. [26] further related the high viscosity of aged powder to surface roughness. There is minimal information on the dimensional accuracy of reused PA11 powder during powder bed fusion laser sintering, which further strengthens the need for this work. 

The main objective of this work is to investigate the effect of reusing PA11 powder in a PBF-LS process on the dimensional accuracy, physical properties, crystallinity and tensile properties of the sintered part. Using a Formlabs Fuse 1 3D printer (CREAT3D, Wokingham, UK), tensile test and density samples were first additively manufactured using 100% virgin PA11 powder. The ratio of used powder to unused powder and its associated weight were analysed. The density cubes were used for density measurements, following which the tensile test pieces were tested using a Universal tester. Pieces of the sintered density cubes were analysed, using differential scanning calorimetry (DSC) to analyse crystallinity. The remaining powder in the chamber was reused up to 10 times, following which the mentioned tests were repeated. The effect of reusing the PA11 powder in PBF-LS up to 10 times was related to the crystallinity and tensile properties. 

## 2. Materials and Methods

### 2.1. Powder Bed Fusion Laser Sintering of Polyamide 11

A Formlabs Fuse 1 3D printer (CREAT3D, Wokingham, UK) was used. It has a build volume of 16.5 cm × 16.5 cm × 30 cm and prints at a layer thickness of 110 µm, with an Ytterbium fibre laser (1065 nm Wavelength; 10 W) of 200 µm spot size. It works with Preform Software, which is supplied with the 3D printer. 

The polyamide 11 powder used in this paper was supplied by CREAT3D (Wokingham, UK) and possessed the mechanical properties listed in Table 1 (obtained from the manufacturers, Formlabs, [27]), which is the baseline for comparison in this work. The powder also had a moisture content of 0.37%, and printed samples with this powder were validated as non-cytotoxic, non-irritant and a non-sensitiser, in accordance with ISO 10993-1 [28]. This means PBF-LS PA11 is safe to use as a medical device that comes into contact with the skin. 

**Figure 1:**
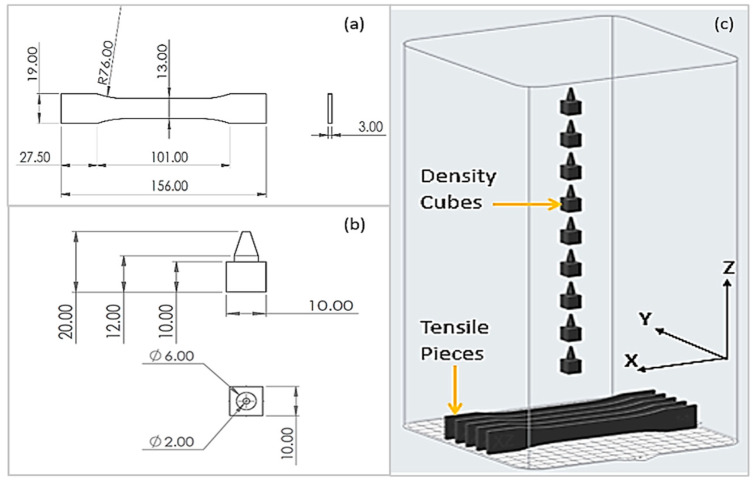
Shape and dimension of (**a**) tensile test sample and (**b**) density cubes; (**c**) location of tensile test samples and density cubes in Fuse 1 printer’s build chamber.

Five tensile test pieces, with dimensions as shown in Figure 1a, were printed in the XZ direction, and density cubes (Figure 1b) were printed top of each (Figure 1c) other, to increase the Z height and, therefore, the amount of powder required to complete the build. The Formlabs Fuse 1 is a closed system and does not allow for the modification of the process parameters. During the 3D printing process, a temperature of 193 °C was recorded in the build chamber. 

The same samples were manufactured 10 times. The first build used 100% virgin PA11 powder and was known as B01. Once the 3D printed parts were removed from the build, the unfused powder from the first build was sieved using a Sift 300 Sieve with Fuse Sift (CREAT3D, Wokingham, UK) and used in the second build. The second build (B02) used 100% recovered powder (no fresh powder added) from B01. The third build used 100% powder recovered from B02, hence labelled B03. This was repeated up to B10. 

### 2.2. Polyamide 11 Powder Usage Analysis

Since, in each build following B01, 100% of the used powder was used, the quantity of the powder input reduced from B01 (6 Kg) to B10 (0.5 Kg). The percentage of fused powder to total powder used in a specific build and the amount of powder wasted per unit of powder recovered were measured, to correlate the effect of the number of reuses to the amount of powder being recovered in each build. The weight of the built parts per unit of fused powder in the build was also measured, to confirm whether the number of reuses affects the density of the sample built. 

### 2.3. Dimensional Accuracy Measurement

A FARO Edge Scan Arm HD (FARO Technologies UK Ltd., Rugby, UK), with an accuracy of ±35 µm and a scan rate of 45,120 points/s, was used to three-dimensionally scan the tensile test pieces. Each specimen was positioned in its print orientation when scanned, e.g., the XZ plane, as shown in Figure 1. Two scans were used to capture the full geometry of the specimen; the main scan captured most of the surfaces, then the specimen was rotated 180° to scan the bottom surface. The 3D scan of the specimen was post-processed, using Polyworks Metrology Suite 2019 software (3DScanners, Barford, UK), to remove any erroneous data points and fill holes in the polygonal mesh. It was then aligned to the reference CAD file, using the best-fit data to reference the alignment.

A data colour map was used to compare the measured 3D scan with the nominal CAD file and illustrate the deviation. Standard calliper gauges were created to measure the gauge width and thickness of each specimen and to calculate the deviation between the nominal CAD file and measured 3D scan data.

### 2.4. Density Measurements 

The density cubes were used to measure the density of the PBF-LS PA11 parts, from B01 (100% virgin powder) to B10. 

For the density measurement, the weight of the individual density cubes was measured in air, *W_A_*, using a high-performance analytical balance (Balance XPR, Mettler Toledo, Loughborough, UK). Their weights were then measured in isopropyl alcohol, *W_B_*. Using the Archimedes principle, the density, ρ, of the cubes was calculated using Equation (1):

(1) $$\rho =\frac{W_{A}}{\left(W_{A}-W_{B}\right)}(\rho_{O}-\rho_{L})+\rho_{L}$$

where ρ_O_ is the density of isopropyl alcohol (0.78977 g/cm^3^ at 19.5 °C) and ρ_L_ is the density of air (0.0012 g/cm^3^) (Balance XPR Datasheet, Mettler Toledo, Loughborough, UK).

For each build, three density values were obtained (*n* = 3). The average of the three was calculated and presented as the average ± S.E.M (standard error of the mean). A one-way ANOVA was performed to analyse the differences between the density data at a 95% confidence interval.

### 2.5. Thermal Properties

To quantify their thermal properties, differential scanning calorimetry (DSC) tests were performed on the PA11 specimens (B01, B03 and BA10) using a DSC4000 (Perkin Elmer, Beaconsfield, UK). DSC specimens, with weights of 6 ± 1 mg, were exposed to a temperature ramp from 25 °C to 250 °C, increasing at a rate of 10 °C/min. They were then cooled down, from 250 to 25 °C, at the same rate [20]. Under a nitrogen flow rate of 40 mL/min, three sets of measurements were obtained. The melting enthalpy of the specimens was calculated by integrating the endothermic peak using the TA instruments Pyros software V13.3.2.0030, and the crystallinity, X_c,_ was calculated using Equation (2):

X_c_ = (ΔH_m_/ΔH^0^_m_) × 100% (2)

where ΔH_m_ is the melting enthalpy calculated from the endothermic peak (J/g) and the melting enthalpy for a 100% crystalline PA11 matrix is denoted by ΔH^0^_m_ (theoretical ΔH^0^_m_ = 226.4 J/g) [20]. The average of the three data per sample were calculated and presented as the average ± S.E.M (standard error of the mean). A one-way ANOVA was performed to analyse the differences between the density data at a 95% confidence interval (CI).

### 2.6. Tensile Testing

The tensile testing was undertaken with respect to ASTM 638-14 (the standard test method for the tensile properties of plastics), using a Shimadzu universal tester, AG-X (Shimadzu, Milton Keynes, UK). At a humidity of 39%, the test was performed up to failure, using a load cell of 100 kN at a speed of 5 mm/min and an Epsilon 3542 axial extensometer (±25 mm). The force and displacement data collected were used to calculate the engineering stress and strain, following which the average tensile modulus, ultimate tensile strength, yield strength and breaking strain were calculated for each build. 

For each build, five values were obtained for each property measured (*n* = 5). The average of the five was calculated and presented as the average ± S.E.M (standard error of the mean). A one-way ANOVA was performed to analyse the differences between the density data at a 95% confidence interval.

## 3. Results

### 3.1. Powder Bed Fusion Laser Sintering of Polyamide 11

**Figure 2:**
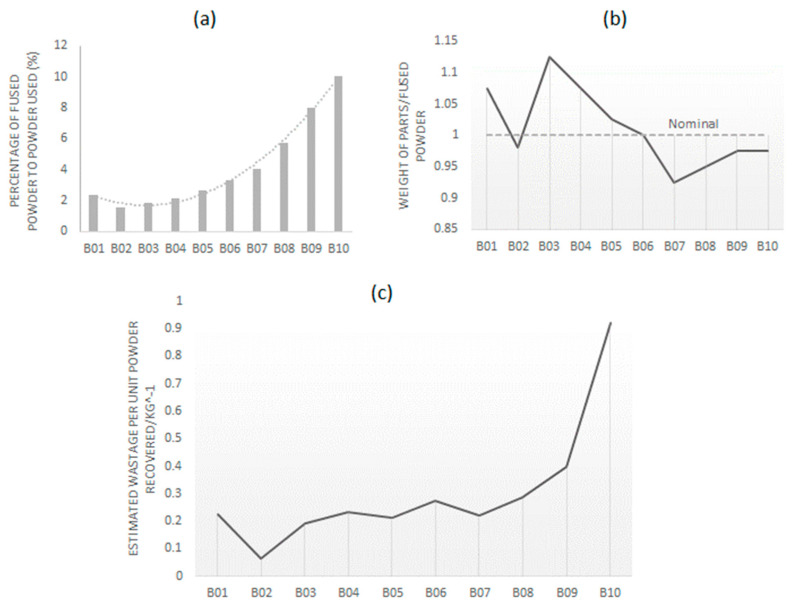
Usage of PA11 powder during the PBF-LS process from B00 to B09; (**a**) percentage of fused powder to the amount of PA11 powder used; (**b**) weight of the 3D-printed parts as a ratio of the weight of PA11 powder fused and (**c**) estimated waste of PA11 powder per unit of mass of powder recovered.

The percentage of fused powder to total powder (fused + unfused powder) used for B01–B10 had a polynomial increase, as shown in Figure 2a. When considering 100% reuse of powder in PBF-LS, the percentage of fused powder (F) can be related to the number of times the powder has been used (N), using the following equation:

F = aN^2^ + bN + c (3)

where a, b and c are fitting constants unique to the type of powder used in the PBF-LS process. For the PBF-LS of PA11, the values of a, b and c are 0.164, 0.955 and 3.091, respectively. 

The weight of all the parts printed from one build did not always match the weight of the fused powder. From B01 to B06, the weight of the samples was higher than that of the fused powder, and it was lower from B07 to B10, as shown in Figure 2b. The higher weight was associated with the polycondensation of the PA11 during the PBF-LS process, which has been observed by Esposito et al. [18]. The increase in weight was associated with the post-polymerisation condensation and associated humidity. The lower weight was associated with the shrinkage of PBF-LS polyamide parts [30]. For every unit of PA11 powder recovered for the next build, there was an increasing amount of waste from B01 to B10. 

### 3.2. Dimensional Accuracy

**Figure 3:**
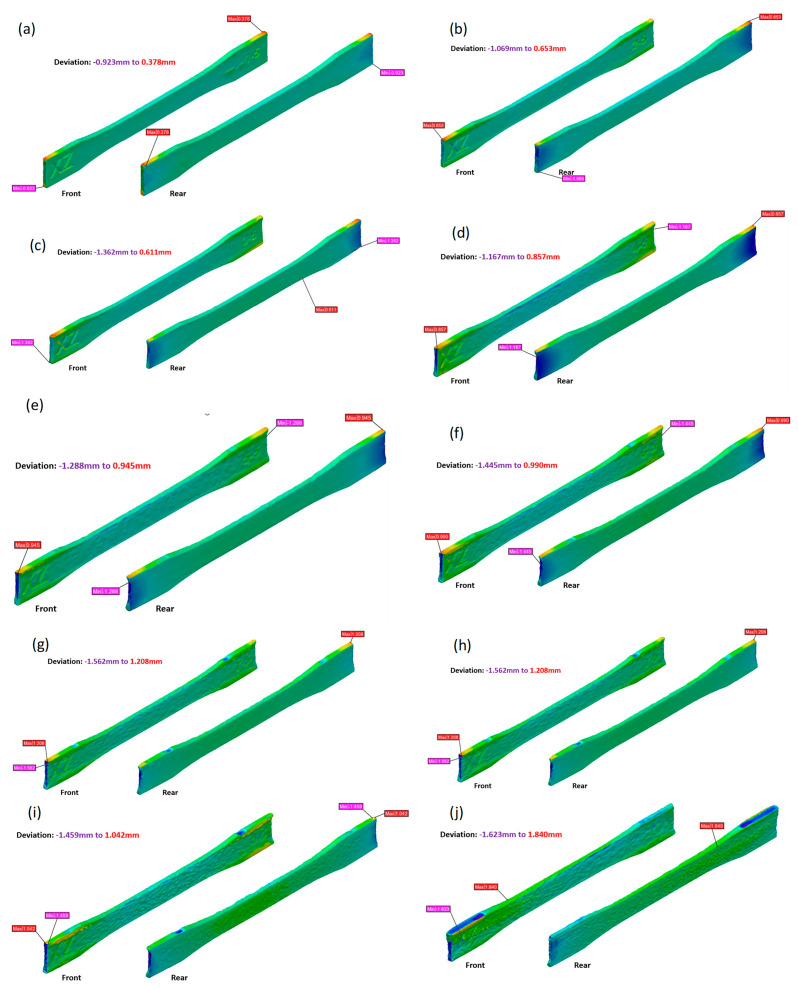
Colour map showing deviation from CAD model for (**a**–**j**) B01 to B10, respectively.

Up to sample 5 (B05; Figure 3e), the letters XZ and sample number could be read from the surface. On a macroscopic level, the increasing unevenness of the surface from B05 to B10 reduced the visibility of the letter and number engravings on the surface. The coarser surface observed with the increasing number of reuses of PA11 powder was related to the higher viscosity and melting point of reused polyamide powders, which therefore remained unmolten on the surfaces of the 3D-printed parts [5]. The high viscosity of the reused PA11 powder resisted the flow of the molten powder during the sintering process [26]. This coarse surface has been observed by other researchers as a result of using an PBF-LS process and has been associated with an ‘orange peel’ effect [25]. 

**Figure 4:**
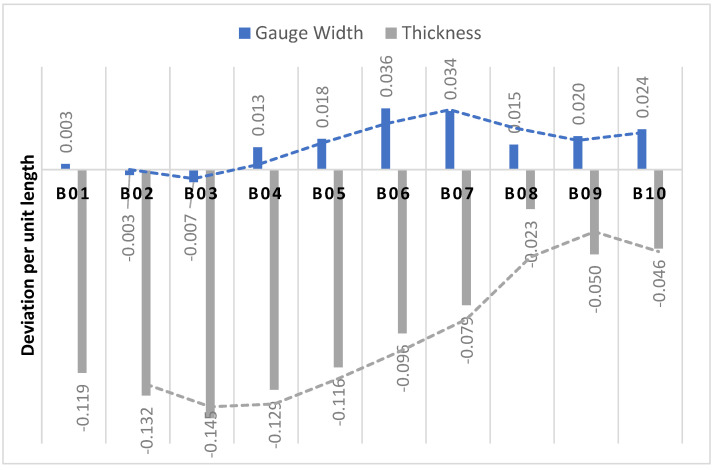
Deviation per unit length in Gauge Width and Thickness of tensile test pieces during builds B01–B10.

The colour maps in Figure 3 represent the deviation of the PBF-LS PA11 samples from the nominal CAD file. The edges and corners experienced more deviation from the CAD compared to flat surfaces, with the deviation values’ comparison depicted in Figure 3. The gauge width represented the ZX build plane, while the thickness represented the XY build plane. The thickness of samples was mostly smaller than the CAD thickness, compared to the gauge width, which was mostly larger than the CAD width, as shown in Figure 4. This indicates shrinkage dominating the thickness compared to the condensation process, and vice versa for the gauge width. It was also related to the ratio of the length to height, which was 7.8:1 for the gauge section and 0.2:1 for the thickness section. A low scan length is known to increase shrinkage along the X axis during the PBF-LS process [24]. This has been associated with volume shrinkages, due to densification and phase transition during the sintering and cooling processes [31]. The low thickness (3 mm) of the test samples being built in the XZ direction resulted in the scan length being only 3 mm. Consequently, shrinkage was at the maximum along the scan length, and, thus, so was the thickness.

**Figure 5:**
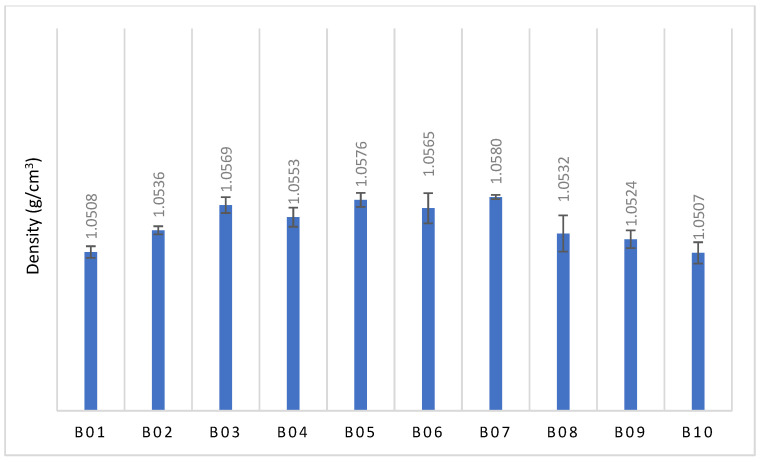
Density of the PA11 cubes from B01 to B10.

The deviation per unit of length for the gauge width was minimal, compared to that of the thickness. The thickness deviation increased from B01 to B03 and then kept decreasing for the remaining builds. From B01 to B03, the PA11 powders were exposed to an elevated temperature for a short time, resulting in few particles with a high melting point. In those builds, there was still a homogeneous distribution of powder particles [32], which allowed the shrinkage effect to dominate, due to the small scan length for the thickness section. From B03 to B10, there was an increasing number of PA11 particles with a high melting point and low viscosity [5], which resulted in the non-homogeneous distribution of the particles for the layer-by-layer process. This accounted for the uneven surface of the gauge section, and this process dominated the effect of material shrinkage for thickness, which resulted in a reduction in deviation from the nominal. This was confirmed by the decrease in density after B07, as shown in Figure 5. The increase in density from B01 to B03 was associated with the increase in molecular weight, due to the polymerisation and cross-linking reactions during the PBF-LS process. The process increased the molecular chains, resulting in an increase in molecular weight and, hence, density [17]. 

### 3.3. Thermal Properties

**Figure 6:**
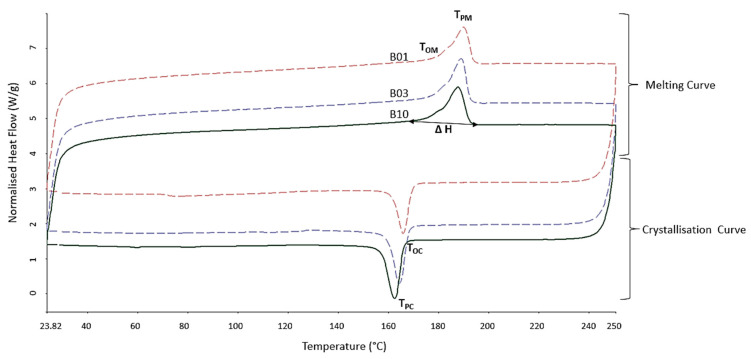
Sample DSC thermograms of B01, B03 and B10.

From the DSC thermograms obtained during the DSC analysis, as shown in Figure 6, the onset melting point (T_OM_), peak melting point (T_PM_), onset crystallisation point (T_OC_), peak crystallisation point (T_PC_) and melting enthalpy (ΔH) were obtained. The crystallinity was then calculated and is presented in Table 2. For all the samples tested, the melt temperatures (onset and peak) were higher than that of the crystallisation temperatures (~25 °C). A delay in crystallisation prevents residual stress from building up, resulting in less distortion due to the sintering process [9]. However, a higher temperature difference is associated with PA12 (~40 °C) [9], which could be associated with more distortion in PBF-LS PA11 parts compared to PBF-LS PA12 parts.

The T_OM_, T_PM_ and X (crystallinity) for B01, B03 and B10 were significantly different from each other (*p* = 0.000014 (T_OM_), 0.000005 (T_PM_), and 0.0003 (X) at 95% CI). There was a decrease in X from virgin PA11 to the third use, following which there was an increase to the tenth use (B10). Dadbakhsh et al. [5] made a similar observation during the PBF-LS of PA12 reused powder, which was attributed to crystal growth caused by a post-condensation phenomenon. To further understand at which point the crystallinity for PA11 samples started to increase after B03, DSC was performed on B04. As shown in Table 2, the crystallinity of B04 was significantly higher than B03 (*p* = 0.01) and slightly lower than B10 (*n* = 3; CI = 95%; *p* = 0.04). 

The onset of crystallisation and the peak crystallisation temperature decreased after the fourth use (B04) (*p* = 0.0005) of PA11 powder for the process of PBF-LS. This agrees with what was observed for PA12 during its repeated use [5] and was associated with longer polymer chains post-condensation. Decreases in T_OC_ and T_PC_ have been associated with the ageing of such polyamides [2]. Hence, after the fourth use of PA11 in the Fuse1 Formlabs printer, the powder exhibited ageing characteristics. 

### 3.4. Mechanical Properties

#### 3.4.1. Ductility

**Figure 7:**
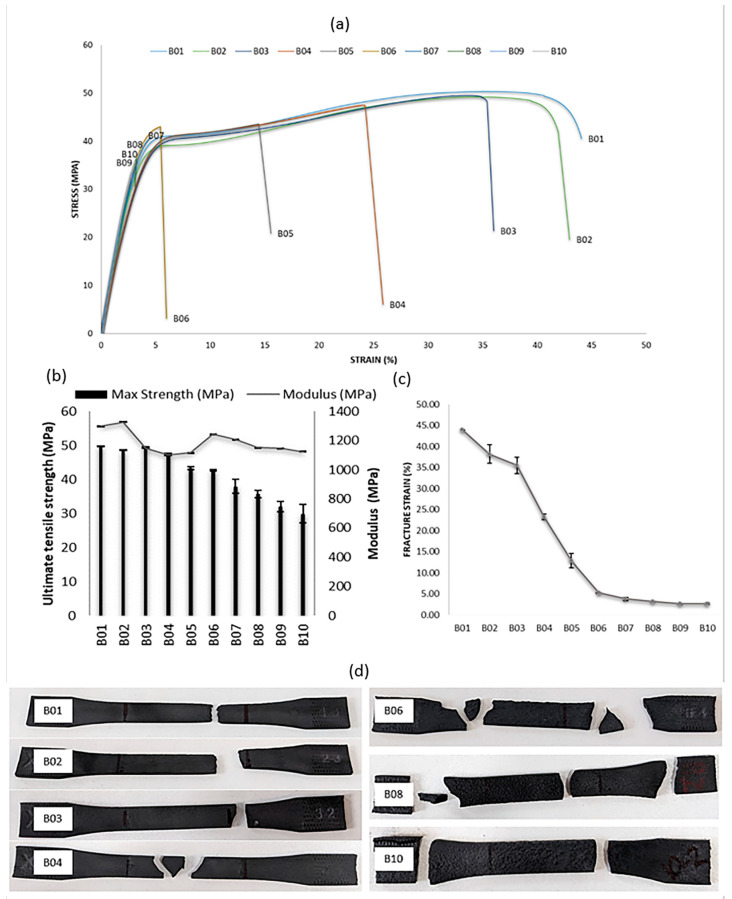
Tensile test results for B01–B10. (**a**) Typical stress–strain curve from each build; (**b**) average ultimate tensile strength and tensile modulus; (**c**) breaking strain of samples from B01 to B10; and (**d**) typical fractured tensile samples for B01–B04, B06, B08 and B10.

Figure 7a shows the typical stress–strain curve obtained from tensile test samples from B01 to B10. B01 to B05 exhibit a linear elastic behaviour, with smooth hardening, until failure. PA11 is a semi-crystalline polymer, consisting of orderly arranged polymer chains (crystalline regions) and randomly arranged polymer chains (amorphous regions). When a tensile load is applied on a tensile test sample, the uniformly arranged polymer chains in the crystalline region resist deformation, while the irregularly arranged chains slide easily. This is reflected by the stress–strain curve for B01, highlighting its higher ductility. This was associated with the necking observed in Figure 7d. When PA11 powder is reused, the polymerisation and cross-linking results in longer complex molecular change, and, hence, more amorphous regions. This is expected to increase the intermolecular mobility, resulting in the higher ductility of B02 onwards. However, the opposite was observed, i.e., the ductility decreased, when the powder was reused. Similar observations have been made for reused PA12 [5], which were related to the weaker bonding and coalescence in reused PA12 powder. From B05 to B10, an increasing brittle behaviour was observed, which was due to an increase in the number of complex polymer chains, leading to a high viscosity and unmolten particles creating more defects in the build. Unmolten powder may also play a role in the nucleation and propagation of cracks in the PA11 samples, leading to a decrease in ductility [17]. This brittleness, identified in Figure 7a, is reinforced by Figure 7d, which shows the increased number of pieces in which the tensile samples fractured. 

#### 3.4.2. Tensile Strength

The average ultimate tensile strength of B01, B02 and B03 was 49.76 ± 0.004, 48.61 ± 0.015 and 49.54 ± 0.008 MPa, respectively, as shown in Figure 7b. The UTS agrees with the values provided by the supplier, as shown in Table 1. Although there was powder reuse from B01 (virgin powder) to B03, there was no change in tensile strength from B01 to B03 (*n* = 5; CI = 95%; *p* = 0.2951). In the Fuse 1 printer, the PA11 powders were exposed to a temperature of 193 °C, which resulted in larger molecule formation post-polymerisation and cross linking. This has been associated with an enhancement of the intermolecular forces [17], which maintained the UTS from B01 to B03. Nonetheless, the UTS decreased significantly from B03 to B04 (*n* = 5; CI = 95%; *p* = 0.0006) to B10. Every use and reuse of the PA11 powder during the PBF-LS process caused the powder to be exposed to a high temperature repeatedly. The powders that had been reused many times, in this case from the third reuse, had a higher molecular weight following the condensation and polymerisation processes, which resulted in a higher melt viscosity [33] compared to those powders in B01 to B03. Additionally, residual porosity was a common phenomenon during the PBF-LS of polymers [34]. Reusing PA11 powders more than twice (without mixing them with virgin powder) increased the porosity, and, hence, the defects, within the PBF-LS parts. This, in turn, reduced the tensile strength of the reused PA11 parts. Similar observations have been made for PA2200 [17], whereby reusing the PA2200 powder twice on its own, started to reduce the UTS. The decrease in UTS from B04 to B10 was also associated with an increase in crystallinity, as shown in Table 2. 

#### 3.4.3. Tensile Modulus

There was no difference (*n* = 5; CI = 95%; *p* = 0.2529) between the tensile modulus of B02 (1327 ± 11.8 MPa) and B01 (virgin PA11; 1298 ± 16.1 MPa). However, the modulus decreased significantly to 1149 ± 27.9 MPa for B03 (*n* = 5; CI = 95%; *p* = 0.003), and, from B03 to B10, there was no significant change in modulus (*n* = 5; CI = 95%; *p* = 0.2302). In B01 and B02, there were stronger intermolecular forces, which resisted movement during tensile loading. It should be noted that the modulus of B01 was different from the ones provided by the manufacturer, as shown in Table 1 (1.6 GPa). This was related to the build direction used for testing by the manufacturers, which was in the XY direction, while, in this work, the ZY direction was used. The reduction in modulus from B03 was associated with the weakening intermolecular forces and the increases in porosity and defects in the 3D-printed parts, due to the sintering process [17,34]. 

#### 3.4.4. Yield Strength

The yield strength was calculated at 0.2% plastic strain. From B05 to B10, no yield strength (YS) data are available, due to the brittle behaviour of the PBF-LS PA 11 parts. From B01 (YS = 19.49 ± 0.74 MPa) to B04 (YS = 17.47 ± 1.11 Mpa), there were no significant differences in yield strength (*n* = 5; CI = 95%; *p* = 0.2279). Up to the fourth use (B04), irrespective of the number of reuses, the stress that could be withstood by PA11, before plastic deformation was observed, was the same. The yield strength was related to the ductility of the respective samples. 

#### 3.4.5. Fracture Strain

The fracture strain of the virgin PA11 samples (B01) was significantly higher, at 43.95%, than any reused PA11 samples (*n* = 5; CI = 95%; *p* = 0.0209). The fractured B01 samples, depicted in Figure 7d, confirmed the high strain due to necking before fractures, identified by the narrowing of the gauge width closer to the fracture site. The fracture strain for B02 and B03 was similar (*n* = 5; CI = 95%; *p* = 0.4624), after which there was a significant decrease in the fracture strain from B03 to B05 (*n* = 5; CI = 95%; *p* = 0.0021). During the reuse of the PA11 powders, the polymer chains became more complex, with increasing molecular weights. This reduced the ability of polymer chains to undergo plastic deformation, hence reducing the fracture strain when reused. It was also observed that there was no change in the elongation at break from B05 to B10 (Figure 7c). The reduction in ductility observed from B05 to B10 in Figure 7a was associated with the respective fracture strain and porosity, due to PBF-LS [34]. In these samples, the tensile stress exceeded the applied stress, resulting in the formation of defects/cracks in the samples at more than one location, as shown in Figure 7d. Thus, there were fractures before plastic deformation was observed. Similar observations have been made for PA2200 after the fourth reuse [17].

## 4. Discussion 

Polyamide 11 is a semi-crystalline polymer with both regularly and irregularly arranged polymer chains. During the powder bed fusion laser sintering process, the powders are fused together, using a laser source in the presence of nitrogen atmosphere. The unused powder is thus exposed to a high temperature, although the presence of nitrogen prevents the thermo-oxidative degradation of the powder [35]. Nonetheless, when the unused powder undergoes the PBF-LS process again, the mechanical and thermal properties of the resulting samples have been observed to change, with accompanying changes in dimensional accuracy. 

When using a virgin feedstock, the PBF-LS process generated PA11 samples with 21.8% crystallinity, which is close to that of compression-moulded PA11 (16%) [21]. Hence, the Formlabs Fuse 1 printer can manufacture parts with a similar crystallinity to traditionally manufactured PA11. 

From B01 (100% virgin powder—1st use) to B03 (100% third use of powder), there was minimal deviation of the gauge length from the nominal and minimal changes in the tensile properties. This was associated with the increase in density of the PBF-LS parts (Figure 5) and, hence, an increase in the molecular weight. At a large molecular weight, the polymer chains are long and more complex, with stronger bonding to each other. Nonetheless, a decrease in crystallinity was observed from B01 to B03 (21.8% to 19.4%), which has been shown to decrease the mechanical properties of polyamides, such as PA12 [36]. PA12 is also a semi-crystalline polymer, with its sintered part exhibiting about 47% crystallinity when 100% virgin powder is used [16]. When reused, the crystallinity decreased to 44%. Chen et al. [16] also observed a more significant decrease in crystallinity from 47% to 23% for PA6. However, in comparison, the percentage of the crystalline phase in PA11 is lower than in PA12 and PA6. Therefore, the reduction in mechanical properties observed when PA12 is reused [36] was not reflected in PA11 in this work. When PA11 powder was used in a PBF-LS process and the remaining powder was reused again twice, although there was a decrease in crystallinity following each use and reuse up to B03, the tensile strength and modulus did not degrade. This meant that the lower ratio of crystalline to amorphous regions in PBF-LS PA11 prevented the crystallinity from dominating intermolecular bonding in the latter, and, hence, the mechanical properties. 

From B04 to B10, an increase in crystallinity was observed to a level higher than that of B01 (virgin powder). The repeated heating and cooling detangled the irregularly arranged polymer chains and arranged them more regularly in the third (B03) and fourth (B04) use of the PA11 powder. Nonetheless, the percentage crystallinity was still lower than that of PBF-LS PA12, as observed by Chen et al. [16]. Similar to B01–B03, the effect of crystallinity on the tensile properties was not significant in B04 to B10. The significant decrease in tensile strength, modulus, fracture strain and ductility in the reused PBF-LS PA11 (B04 onwards) was thus associated with the increase in molecular weight and surface defects, as identified in the results. 

To further understand the exact mechanism behind the changes in the physical and mechanical properties of PBF-LS PA11, the chemical ageing mechanism of PBF-LS PA11 needs to be further analysed; this will identify the specific change in polymer chains during reuse. Subsequently, the microstructural changes in PA 11 powder and the sintered PA11 during reuse in a PBF-LS process will reflect the chemical changes, by visually distinguishing the polymer chains arrangement in the crystalline and amorphous phases. A study analysing the PA11 powder, its viscosity, rheology and powder surface topology will define the behaviour of the latter during a sintering process. The same analysis needs to be carried out on mixed PA11 powders (both virgin and reused) to understand the effect on refresh rates. 

This paper studied a closed system, whereby the printing parameters could not be modified. Increasing the scanning speed and laser powder have been shown to reduce surface roughness and, hence, increase the dimensional accuracy of PA12 sintered samples [37]. Increasing the build chamber temperature has also been shown to enhance the surface properties, by increasing the density and strength of sintered PA12 [38]. The authors also associated an increased layer thickness with a reduction in warpage and the shrinkage of PA12. Similar observations can be expected for PA11. Hence future work investigating the effect of processing parameters on PBF-LS PA11 is also needed. 

## 5. Conclusions

This paper investigated the effect of reusing PA11 powder during a powder bed fusion laser sintering process (PBF-LS), using a Formlabs Fuse 1 Printer, on the physical and tensile properties of the sintered PA11 parts. The following key observations were made:

(a) Polyamide 11 is a semi-crystalline polymer with about 22% crystallinity, which is lower than that of PA12. Thus, the crystallinity of PA11 does not dominate the tensile properties of PBF-LS PA11.
(b) When using the XZ build direction during a PBF-LS process, lower dimensions of sample thickness than the CAD model were associated with shrinkage along the X- axis, owing to the short scan length for thickness. Longer length sections such as the gauge width had higher dimensions, due to the condensation polymerisation of the PA11 polymer chains during the sintering process.
(c) The physical (density) and tensile (UTS, ductility, modulus and fracture strain) of PA11 is the same or better than virgin powder when reused up to three times (100% reuse, no mixing of virgin powder). from the fourth use, the tensile properties of the sintered PA11 sample degrades significantly with brittleness/ductility being the property being most affected.

This paper provides significant details on the impact of reusing PA11 powder in a PBF-LS process, without being mixed with virgin powder. It helps us in understanding the fundamental of sintering semi-crystalline polymers with a low crystallinity, which can add value to ASTM F3456-22 for medical device additive manufacturing. 

## Figures and Tables

**Table 1:** Mechanical properties of PA 11 samples, printed using the Formlabs Fuse 1 printer and after conditioning at 50% humidity and a temperature of 23 °C [27].

Tensile Property	Value	Method
Ultimate tensile strength	49 MPa	ASTM D 638-14 [29]
Tensile modulus	1600 MPa	
Elongation at break (XY)	40%	

**Table 2:** Thermal properties of B01, B03, B04 and B10.

Build	Onset Melting	Peak Melting	Enthalpy	Crystallisation Onset	Crystallisation Peak	Crystallinity
	T_OM_	T_PM_	ΔH (J/g)	T_OC_	T_PC_	X_m (%) ± S.E.M
B01	182.79	190.15	49.39	169.48	165.39	21.82 ± 0.04
B03	181.89	189.45	43.84	168.45	164.64	19.37 ± 0.13
B04	182.21	189.19	49.45	168.17	165.8	21.84 ± 0.03
B10	180.38	187.5	54.09	166.04	162.15	23.89 ± 0.46

## Data Availability

The data presented in this study are available upon request from the corresponding author.
